# Endoscopic ultrasound-guided fine-needle aspiration for the diagnosis of laryngeal mucosa-associated lymphoid tissue lymphoma

**DOI:** 10.1055/a-2638-5528

**Published:** 2025-07-14

**Authors:** Wenwen Xiao, Yemei Wu, Jiaqi Dai, Haitao Zhao, Mingyao Xu, Hongbo Wang

**Affiliations:** 1 117922 Department of Endoscopy Center, Hubei Cancer Hospital, Tongji Medical College, Huazhong University of Science and Technology, Wuhan, China

This case demonstrates how endoscopic ultrasound-guided fine-needle aspiration (EUS-FNA) can safely and effectively overcome the limitations of traditional biopsy in submucosal laryngeal lesions.

A 61-year-old woman underwent surgical treatment for a left orbital nodule 4 years ago. Postoperative pathology confirmed extranodal marginal zone lymphoma of the mucosa-associated lymphoid tissue (MALT lymphoma). She subsequently received four cycles of cyclophosphamide, doxorubicin, vincristine, and prednisolone (CHOP) chemotherapy and local radiotherapy. One month ago, she underwent laryngoscopy for investigation of throat discomfort. The examination revealed swelling in the left aryepiglottic fold, left vestibular wall, and left laryngeal compartment, with smooth surface mucosa ( Fig. 1 ). Magnetic resonance imaging of the throat showed an abnormal signal in the left piriform fossa, involving the left aryepiglottic fold ( Fig. 2 ).

**Fig. 1:**
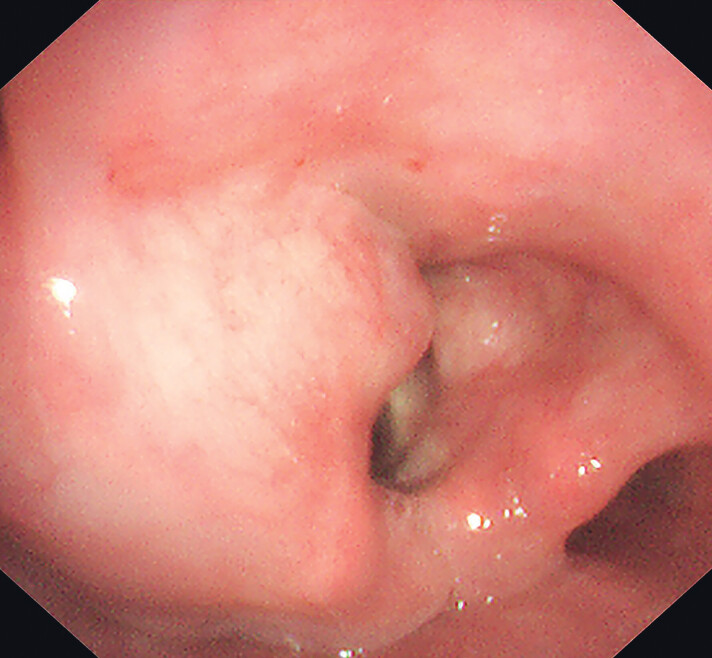
Laryngoscopy showed swelling of the left aryepiglottic fold, left vestibular wall, and left laryngeal compartment, with smooth surface mucosa.

**Fig. 2:**
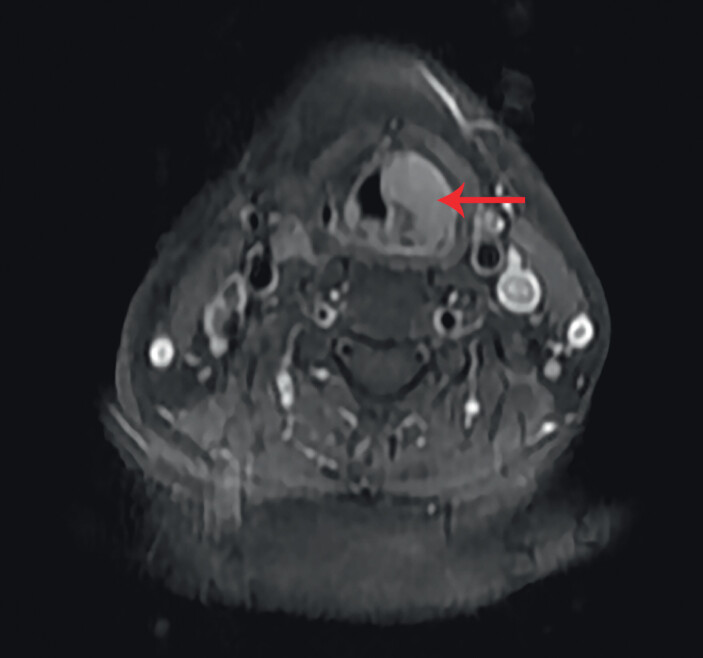
Magnetic resonance imaging revealed an abnormal signal (arrow) in the left piriform fossa involving the left aryepiglottic fold.

Given the smooth mucosal surface of the tumor and the narrow supraglottic space, direct biopsy under laryngoscopy risked a lower diagnostic yield and airway obstruction. Therefore, we opted for EUS-FNA to confirm the diagnosis. EUS revealed a hypoechoic area in the aryepiglottic fold, with clear boundaries and low internal vascularity ( Fig. 3 ). Under real-time Doppler ultrasound guidance (BF-UC260FW; Olympus, Tokyo, Japan), a 21-gauge needle (NA-201SX-4021; Olympus) was used to puncture the lesion ( Video 1 ). The procedure caused minimal bleeding, and histopathology confirmed MALT lymphoma ( Fig. 4 ). Finally, the patient received repeat chemotherapy and local radiotherapy.

**Fig. 3:**
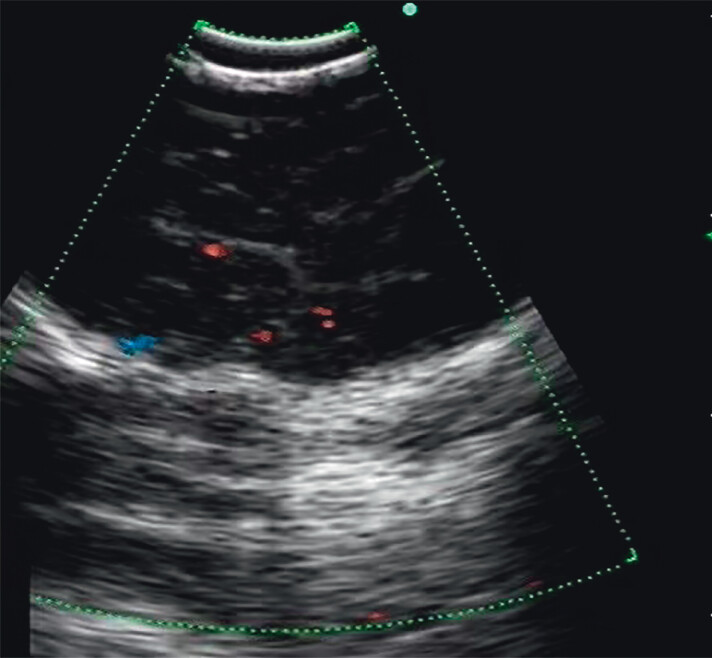
Endoscopic ultrasound revealed a hypoechoic area in the left aryepiglottic fold, with well-defined boundaries and low internal vascularity.

**Fig. 4:**
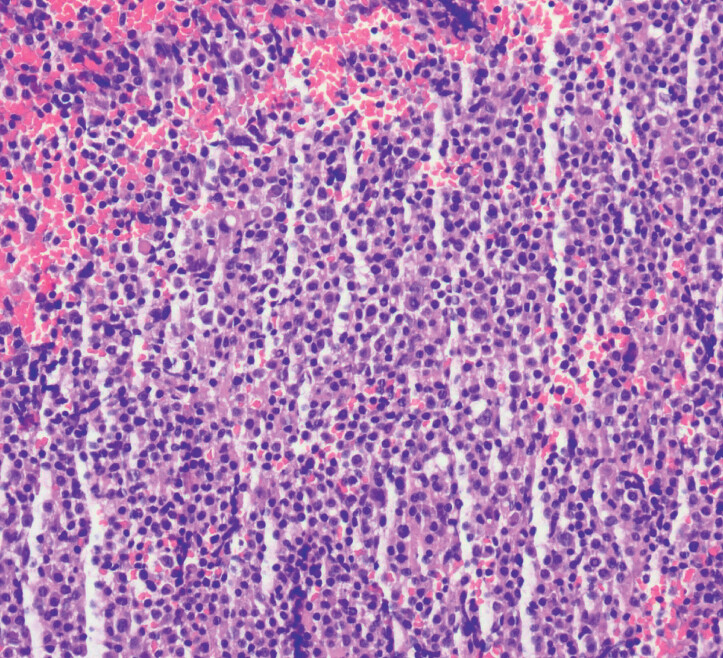
Hematoxylin and eosin staining confirmed mucosa-associated lymphoid tissue lymphoma.

Endoscopic ultrasound revealed a hypoechoic area in the left aryepiglottic fold, with clear margins and low internal blood flow. Under real-time Doppler ultrasound guidance, a 21-gauge needle was used to puncture the lesion.Video 1

MALT lymphoma involving the larynx is relatively rare, and such lesions are typically located partially or entirely beneath the mucosa 1 . Conventional endoscopic biopsy is limited to the mucosal layer, and inadequate sampling depth often renders definitive diagnosis challenging. The application of EUS-FNA to submucosal lesions in the oropharynx is safe 2 ; therefore, it is also suitable for use in the larynx. We present this case to offer new perspectives on the diagnosis and management of submucosal laryngeal lesions. EUS-FNA provides a relatively safe and effective approach for obtaining tissue samples from these lesions.

Endoscopy_UCTN_Code_CCL_1AB_2AB

## Notes

Endoscopy E-Videos https://eref.thieme.de/e-videos
*E-Videos* is an open access online section of the journal *Endoscopy* , reporting on interesting cases and new techniques in gastroenterological endoscopy. All papers include a high-quality video and are published with a Creative Commons CC-BY license. Endoscopy E-Videos qualify for HINARI discounts and waivers and eligibility is automatically checked during the submission process. We grant 100% waivers to articles whose corresponding authors are based in Group A countries and 50% waivers to those who are based in Group B countries as classified by Research4Life (see: https://www.research4life.org/access/eligibility/ ). This section has its own submission website at https://mc.manuscriptcentral.com/e-videos .
